# The Mitochondrial Protease LonP1 Promotes Proteasome Inhibitor Resistance in Multiple Myeloma

**DOI:** 10.3390/cancers13040843

**Published:** 2021-02-17

**Authors:** Laure Maneix, Melanie A. Sweeney, Sukyeong Lee, Polina Iakova, Shannon E. Moree, Ergun Sahin, Premal Lulla, Sarvari V. Yellapragada, Francis T. F. Tsai, Andre Catic

**Affiliations:** 1 Department of Molecular and Cellular Biology, Baylor College of Medicine, Houston, TX 77030, USA; laure.maneix@bcm.edu (L.M.); melanie.sweeney@bcm.edu (M.A.S.); piakova@bcm.edu (P.I.); shannon.moree@bcm.edu (S.E.M.); ftsai@bcm.edu (F.T.F.T.); 2 Huffington Center on Aging, Baylor College of Medicine, Houston, TX 77030, USA; esahin@bcm.edu; 3 Stem Cells and Regenerative Medicine Center, Baylor College of Medicine, Houston, TX 77030, USA; 4 Center for Cell and Gene Therapy, Baylor College of Medicine, Houston, TX 77030, USA; lulla@bcm.edu; 5 Verna and Marrs McLean Department of Biochemistry and Molecular Biology, Baylor College of Medicine, Houston, TX 77030, USA; slee@bcm.edu; 6 Department of Medicine, Baylor College of Medicine, Houston, TX 77030, USA; sarvari.yellapragada@va.gov; 7 Dan L. Duncan Comprehensive Cancer Center, Baylor College of Medicine, Houston, TX 77030, USA; 8 Michael E. DeBakey VA Medical Center, Houston, TX 77030, USA; 9 Department of Molecular Virology and Microbiology, Baylor College of Medicine, Houston, TX 77030, USA

**Keywords:** ubiquitin-proteasome system, mitoprotease, multiple myeloma, bortezomib, carfilzomib, drug resistance

## Abstract

**Simple Summary:**

Multiple myeloma is the second most common cancer of the blood system in the US. Despite new therapies, a cure remains elusive, and current drugs inevitably become ineffective due to various resistance mechanisms. A frontline clinical strategy is the inhibition of the proteasome, the main cellular machinery that degrades proteins in the cytosol and nucleus. Mitochondria are organelles that contain their own set of proteases for protein degradation. Surprisingly, proteases inside mitochondria are also capable of processing proteins normally found outside of these organelles. In this study, we provide evidence that the mitochondrial protease LonP1 can compensate when the proteasome is inhibited and that increased levels of LonP1 confer partial resistance against proteasome inhibitors in multiple myeloma.

**Abstract:**

Multiple myeloma and its precursor plasma cell dyscrasias affect 3% of the elderly population in the US. Proteasome inhibitors are an essential part of several standard drug combinations used to treat this incurable cancer. These drugs interfere with the main pathway of protein degradation and lead to the accumulation of damaged proteins inside cells. Despite promising initial responses, multiple myeloma cells eventually become drug resistant in most patients. The biology behind relapsed/refractory multiple myeloma is complex and poorly understood. Several studies provide evidence that in addition to the proteasome, mitochondrial proteases can also contribute to protein quality control outside of mitochondria. We therefore hypothesized that mitochondrial proteases might counterbalance protein degradation in cancer cells treated with proteasome inhibitors. Using clinical and experimental data, we found that overexpression of the mitochondrial matrix protease LonP1 (Lon Peptidase 1) reduces the efficacy of proteasome inhibitors. Some proteasome inhibitors partially crossinhibit LonP1. However, we show that the resistance effect of LonP1 also occurs when using drugs that do not block this protease, suggesting that LonP1 can compensate for loss of proteasome activity. These results indicate that targeting both the proteasome and mitochondrial proteases such as LonP1 could be beneficial for treatment of multiple myeloma.

## 1. Introduction

Multiple myeloma is the second most common hematological malignancy in the US [1]. Despite promising new drugs, this cancer remains incurable [2]. Derived from terminally differentiated plasma cells, myeloma cells produce and secrete antibodies at high rates, and their capacity for protein quality control is strained [3]. The proteasome is the endpoint of many protein degradation pathways and represents an Achilles’ heel in this cancer [4,5]. Since their clinical introduction in 2003, proteasome inhibitors have become the first-line treatment for multiple myeloma. Unfortunately, a resistance to treatment inevitably develops [6]. The cause of resistance includes a variety of unrelated mechanisms such as up-regulation or mutation of proteasome subunits, increased multidrug transporter activity, and metabolic changes [7,8,9,10,11,12]. However, in most cases the precise nature of drug resistance remains unknown.

Specific elimination of proteins inside a cell is mainly accomplished by the ubiquitin-proteasome system [4]. This pathway entails several enzymatic steps that act in combination to selectively degrade polypeptides. Proteins that are targeted for removal are covalently modified through ubiquitination. Certain ubiquitin linkages are then recognized by proteasomes, which are large complexes in the cytosol and nucleus that consist of regulatory and core subunits that catalyze the proteolysis of substrate proteins [13]. The ubiquitin-proteasome system represents a first-line target for intervention in multiple myeloma, and several inhibitors of the proteasome are used in the clinic to increase protein stress and eventually destroy multiple myeloma cells [14].

Mitochondria play an important role in multiple myeloma. Given the energy-draining biology of this protein-secreting cancer, mitochondria are the main source of ATP. In fact, while several cancers have been postulated to rewire their metabolism to bypass mitochondria (“Warburg effect”), multiple myeloma has been shown to engage in the opposite “anti-Warburg effect”, in which extracellular lactate is shuttled into cells for mitochondrial consumption [15,16]. Mitochondrial activity has also been shown to increase in multiple myeloma cell lines that have adapted to proteasome inhibition, suggesting a role of these organelles in conferring resistance to therapeutic intervention [10]. Besides their metabolic function, mitochondria have also been shown to facilitate the degradation of cytosolic proteins [17,18,19]. Insoluble protein aggregates that cannot be cleared by the canonical ubiquitin-proteasome pathway can be taken up by these organelles and digested by mitochondrial proteases, some of which still resemble their ancestral bacterial counterparts. Their proteolytic activity may complement the activity of the proteasome as the main cytosolic and nuclear protease [19]. Moreover, some proteasome inhibitors have been shown to also inhibit mitochondrial proteases [20]. It is therefore unclear whether the efficacy of proteasome inhibitors is solely proteasome-related, or whether inhibition of mitochondrial proteases is also clinically relevant.

We hypothesized that the activity of mitochondrial proteases contributes to extra-mitochondrial protein quality control in multiple myeloma and that they promote therapeutic resistance by compensating for proteasome inhibition. To test this hypothesis, we investigated the expression profiles of mitochondrial proteases following proteasome inhibition, analyzed the potency of different proteasome inhibitors in combination with mitochondrial protease inhibitors, and determined the effect of mitochondrial protease expression in vitro and clinically on cancer growth. The results of our study suggest that the essential matrix protease LonP1 is connected to proteasome activity and that it can promote therapeutic resistance against proteasome inhibitors [21].

## 2. Results

### 2.1. Mitochondrial Proteases in Multiple Myeloma

**Figure 1:**
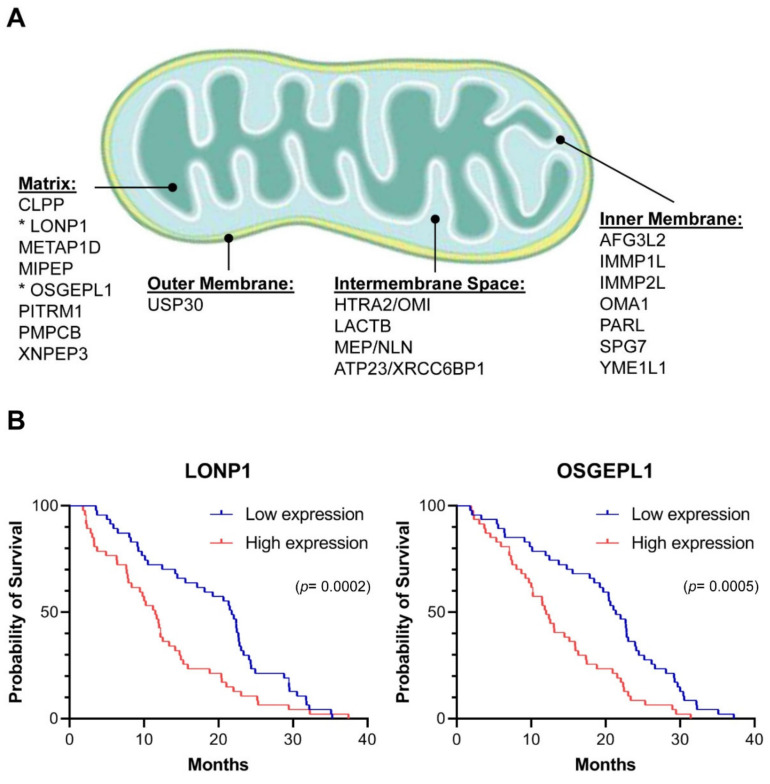
Mitochondrial proteases in multiple myeloma: (**A**) 20 intrinsic proteases have been described in mitochondria [22,23]. Based on their impact on clinical outcomes in multiple myeloma, this study focuses on LONP1 and OSGEPL1 (asterisks); (**B**) The transcript levels of the 20 intrinsic mitoproteases in primary multiple myeloma cells were determined based on [24], and the survival of patients with cancer cells expressing each individual mitoprotease either within the top or the bottom quarter was compared in the cohort receiving bortezomib treatment. Only differential expression of LONP1 and OSGEPL1 showed a significant impact on clinical outcomes, with higher expression correlating with a more aggressive cancer and a 1.9- and 1.8-fold shorter median survival, respectively. No difference was observed in the cohort receiving dexamethasone treatment (not shown). The indicated *p*-values were calculated with the Gehan–Breslow–Wilcoxon test and significance was confirmed with the Mantel–Cox test.

Mitochondria evolved from endosymbiotic proteobacteria and as such contain several bacterial-like proteases [22]. Human mitochondria contain 20 intrinsic proteases (mitoproteases) that are encoded in the nucleus, translated in the cytosol, and targeted to various compartments inside the organelle (Figure 1A) [23]. To examine whether expression levels of these proteases in primary cancer cells are correlated with clinical outcomes, we analyzed pharmacogenomic data from a multicenter study in which the survival of patients with multiple myeloma was correlated with genome-wide transcript levels [24]. The patients in the dataset were either treated with the proteasome inhibitor bortezomib or with dexamethasone. Only two of the twenty mitoproteases showed a significant correlation with cancer survival in the presence of proteasome inhibitor. High expression of LONP1 and OSGEPL1 (O-sialoglycoprotein endopeptidase-like protein 1) in multiple myeloma cells were both associated with more aggressive outcomes and a 1.9- and 1.8-fold shorter median survival, respectively (Figure 1B). Expression levels of these two mitoproteases had no impact on the survival of patients that were treated with dexamethasone (not shown). These results suggest a possible role of high LONP1 and OSGEPL1 expression in resistance to proteasome inhibition.

### 2.2. Regulation of LONP1 and OSGEPL1 Genes in Multiple Myeloma Cells

**Figure 2:**
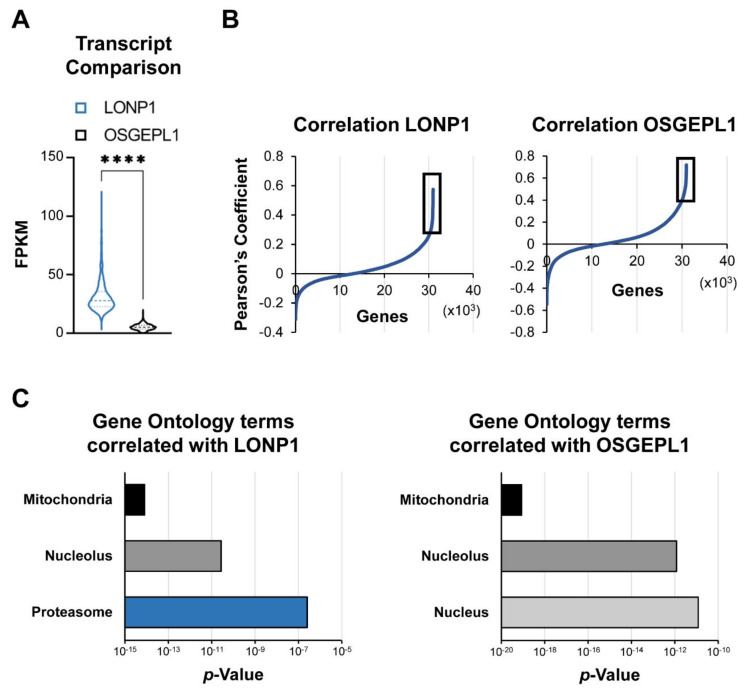
Transcriptional regulon of LONP1 and OSGEPL1: (**A**) Next-generation RNA-sequencing data from multiple myeloma cells of 747 patients [25] show five-fold higher median expression and a four-fold higher interquartile range of LONP1 compared to OSGEPL1. **** *p* < 0.001 determined by two-sided Mann–Whitney U-test; (**B**) We compared genes that most correlate with these two mitoproteases by calculating the Pearson’s correlation coefficient to each of the other 35,134 annotated genes. Defined as significant were correlations above the upper vertex point in the shown waterfall plot (black box). A list of genes correlating with LONP1 and OSGEPL1 mRNA expression is provided in Supplementary Table S1; (**C**) Only LONP1 had significant co-regulation with proteasome subunits, especially with PSMA5, PSMB1, and PSMB2. The *p*-values for the indicated gene-ontology terms enriched within significantly correlated genes were calculated with the functional annotation tool DAVID (https://david.ncifcrf.gov; access date 5 November, 2020) [26,27].

To better understand the transcriptional networks in which LONP1 and OSGEPL1 function, we analyzed the genome-wide co-expression relationships of these two genes in multiple myeloma cells of 747 patients [25]. In primary multiple myeloma cells, LONP1 was more than five-fold higher expressed at the transcript level than OSGEPL1 and, importantly, showed a four-fold higher distribution spread between samples (Figure 2A). We examined the regulatory context of these two genes by analyzing their pairwise Pearson’s correlation coefficients to all other expressed transcripts. Significant co-regulation was defined for genes that had correlation coefficients above those at the ultimate maximal curvature (vertex or knee point) of the waterfall plot (Figure 2B, Supplementary Table S1). Both genes showed co-regulation with nuclear-encoded mitochondrial genes and, interestingly, nucleolar genes. However, only LONP1 displayed co-regulation with proteasomal subunits (Figure 2C). LONP1 expression correlated with a significant number of 20S core and 19S regulatory proteasome subunits: ADRM1, PSMA5, PSMB1, PSMB2, PSMC1, PSMC2, PSMC4, PSMD3, PSMD7, PSMD11, PSMD12, PSMD14, PSME3, PSMG3, RAD23A, and USP14.

**Figure 3:**
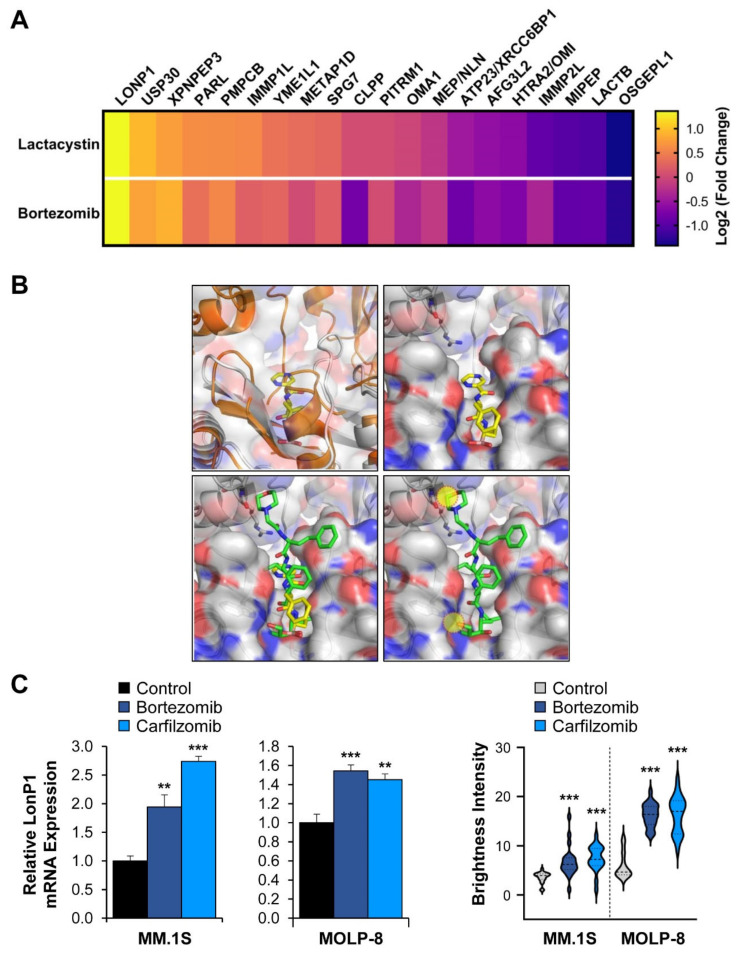

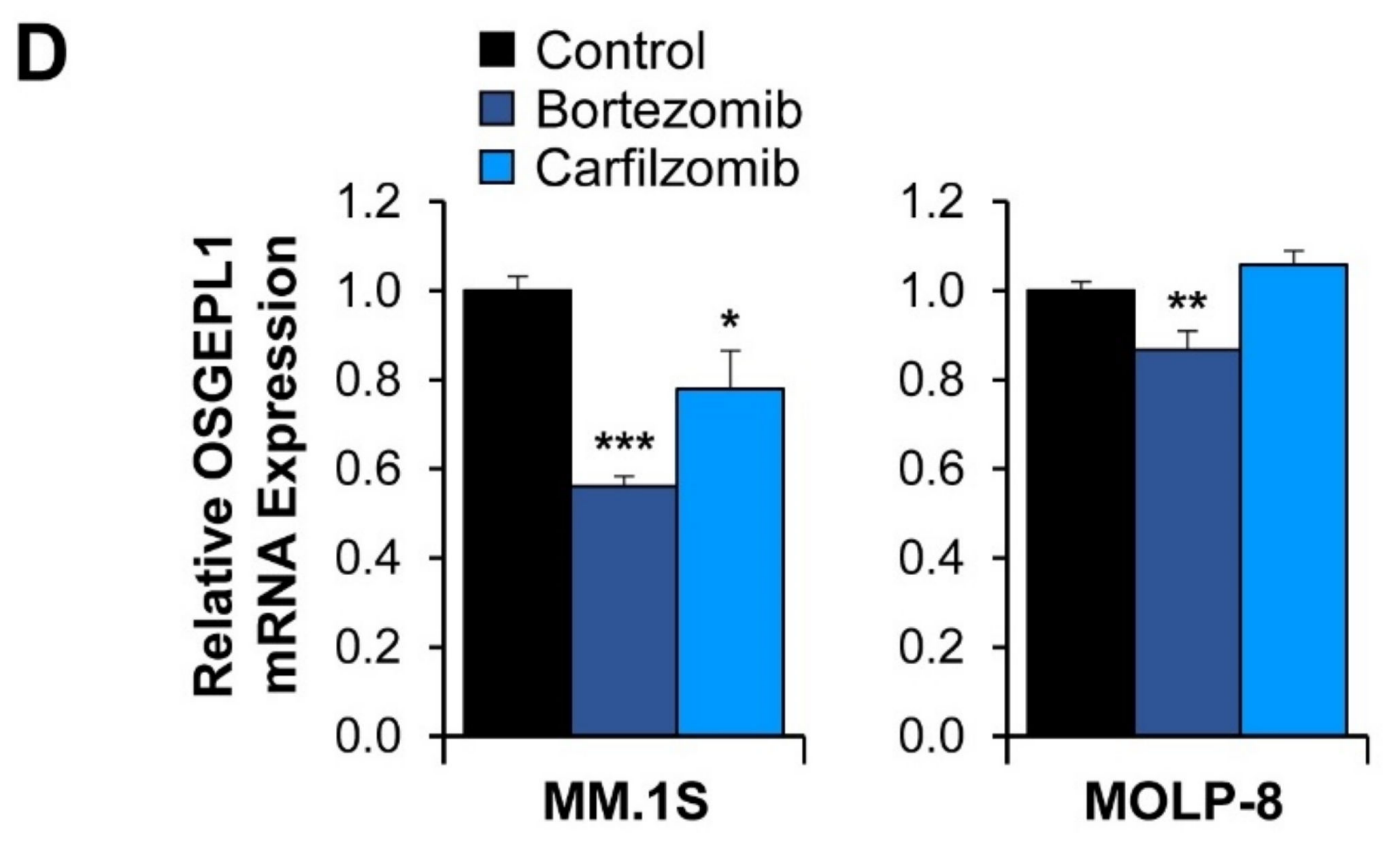
The effect of proteasome inhibition on mitoprotease expression: (**A**) The MM.1S multiple myeloma cell line was treated for six hours with the synthetic proteasome inhibitor lactacystin (6 µM) or bortezomib (60 nM), and RNA expression changes were quantified compared to DMSO control-treated cells by RNA-sequencing. The expression changes of the mitoproteases were plotted from most strongly up- to strongly down-regulated. While LONP1 is highly up-regulated following treatment, OSGEPL1 is down-regulated; (**B**) Structure of the Lon protease with bortezomib. The top left panel depicts the superposition between unliganded human mitochondrial LonP1 (PDB: 7KSM) and *Meiothermus taiwanensis* LonA bound to bortezomib (PDB: 4YPM) [29,30]. Human LonP1 is shown as an orange cartoon with transparent surface. LonA is shown as a grey cartoon with bortezomib as stick model. Because of a steric clash, LonP1 must undergo a conformational change upon drug binding. The top right panel shows a model of bortezomib-binding based on the crystal structure of the LonA complex. Lon is shown as a cartoon and transparent surface with bortezomib shown as a stick model. Residues involved in bortezomib binding are conserved between human LonP1 and bacterial LonA. The bottom left panel shows a predicted model of carfilzomib-binding to Lon. Carfilzomib (PDB: 4QW6.H) was superposed onto bortezomib as seen in the crystal structure of the LonA complex [31]. LonA is shown as a cartoon and transparent surface while carfilzomib (green) and bortezomib (yellow) are shown as stick models. The bottom right panelshows that carfilzomib binding is incompatible with the bortezomib-bound structure and results in steric clashes with Lon, which are indicated by the yellow circles; (**C**) RT-qPCR confirmation of LONP1 up-regulation. Increased LonP1 expression was also observed at the protein level using immuno-fluorescence microscopy (Supplementary Figure S1). Brightness levels of LonP1-specific staining were normalized to DAPI staining and measured in 20 randomly chosen cells. Significantly increased mitochondrial LonP1 staining was observed in MM.1S and MOLP-8 cells following proteasome inhibition. ** *p* < 0.01, *** *p* < 0.001 by unpaired Student’s two-tailed *t*-test; (**D**) We observed OSGEPL1 down-regulation upon treatment of MM.1S and partial down-regulation in MOLP-8 cells with bortezomib and carfilzomib. MM.1S and MOLP-8 cells were treated for six hours with 10 nM or 20 nM bortezomib, respectively. Carfilzomib treatment was performed for six hours at 20 nM. * *p* < 0.05, ** *p* < 0.01, and *** *p* < 0.001 by unpaired Student’s two-tailed *t*-test.

We next sought to investigate how mitochondrial protease expression changes upon protein stress outside of mitochondria. Specifically, we determined how inhibition of the proteasome impacts mitoproteases. If the organelle-specific proteases can indeed compensate for the proteasome, we would expect increased expression of these enzymes upon drug treatment of cells. Using the synthetic proteasome inhibitor lactacystin and the clinically approved inhibitor bortezomib, we observed similar changes in transcript levels of the 20 mitoproteases in the multiple myeloma cell line MM.1S. While OSGEPL1 expression was diminished upon treatment, LONP1 was consistently the most up-regulated mitoprotease in cells treated with both proteasome inhibitors (Figure 3A).

The implication that LonP1 might be involved in shared pathways with the ubiquitin-proteasome system is notable, given that the yeast ortholog of LonP1 (PIM1) can facilitate degradation of cytosolic protein aggregates that are too large for the proteasome to handle [19]. These results therefore suggest that LonP1 might partially compensate for loss of proteasome function by pharmacological blockade. However, a caveat of this interpretation is the possibility that some proteasome inhibitors might inhibit LonP1 directly. The transcriptional up-regulation of this mitoprotease might represent a feedback mechanism to compensate for impaired mitochondrial protein homeostasis, rather than a response to increased protein stress outside of mitochondria. Indeed, both lactacystin and bortezomib have been described to partially crossinhibit LonP1 [20]. However, the clinical proteasome inhibitor carfilzomib has been described as proteasome-specific and does not inhibit LonP1 [28]. A superposition of bortezomib and carfilzomib shows that the latter is incompatible with binding to the predicted bortezomib-binding site of human LonP1 (Figure 3B), consistent with other reports [29]. The fact that multiple myeloma cells treated with carfilzomib showed increased LONP1 expression indicates that the up-regulation of this mitoprotease is a result of proteasome inhibition, and not in response to LonP1 inhibition (Figure 3C and Supplementary Figure S1). OSGEPL1 was down-regulated or unchanged following proteasome inhibition (Figure 3D), making it unlikely that this protease compensates for proteasome inhibition.

### 2.3. The Interplay of LonP1 and Proteasome Activities in Multiple Myeloma Cells

Proteasome inhibition has a wide range of effects and the increased expression of LonP1 could be caused by the general elevation of transcription of stress-response genes. To test whether LonP1 and the proteasome are instead functionally connected, we designed a set of pharmacological experiments to probe the physiologic relationship between these two protease complexes.

**Figure 4:**
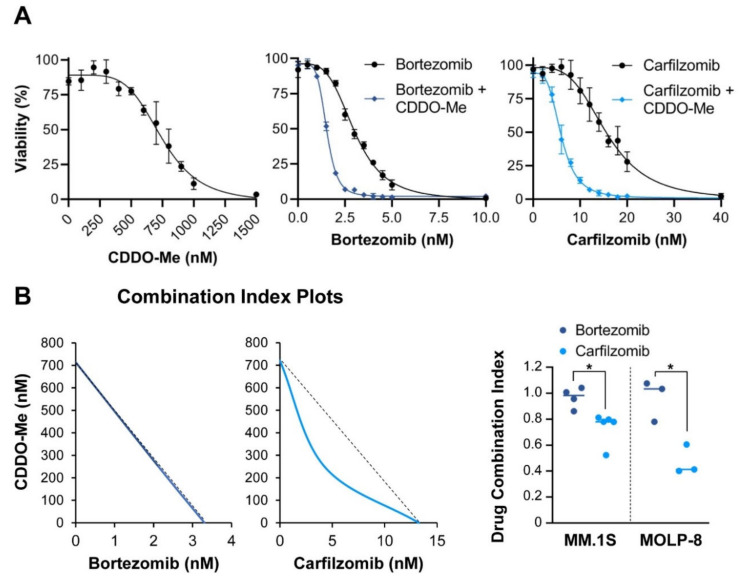

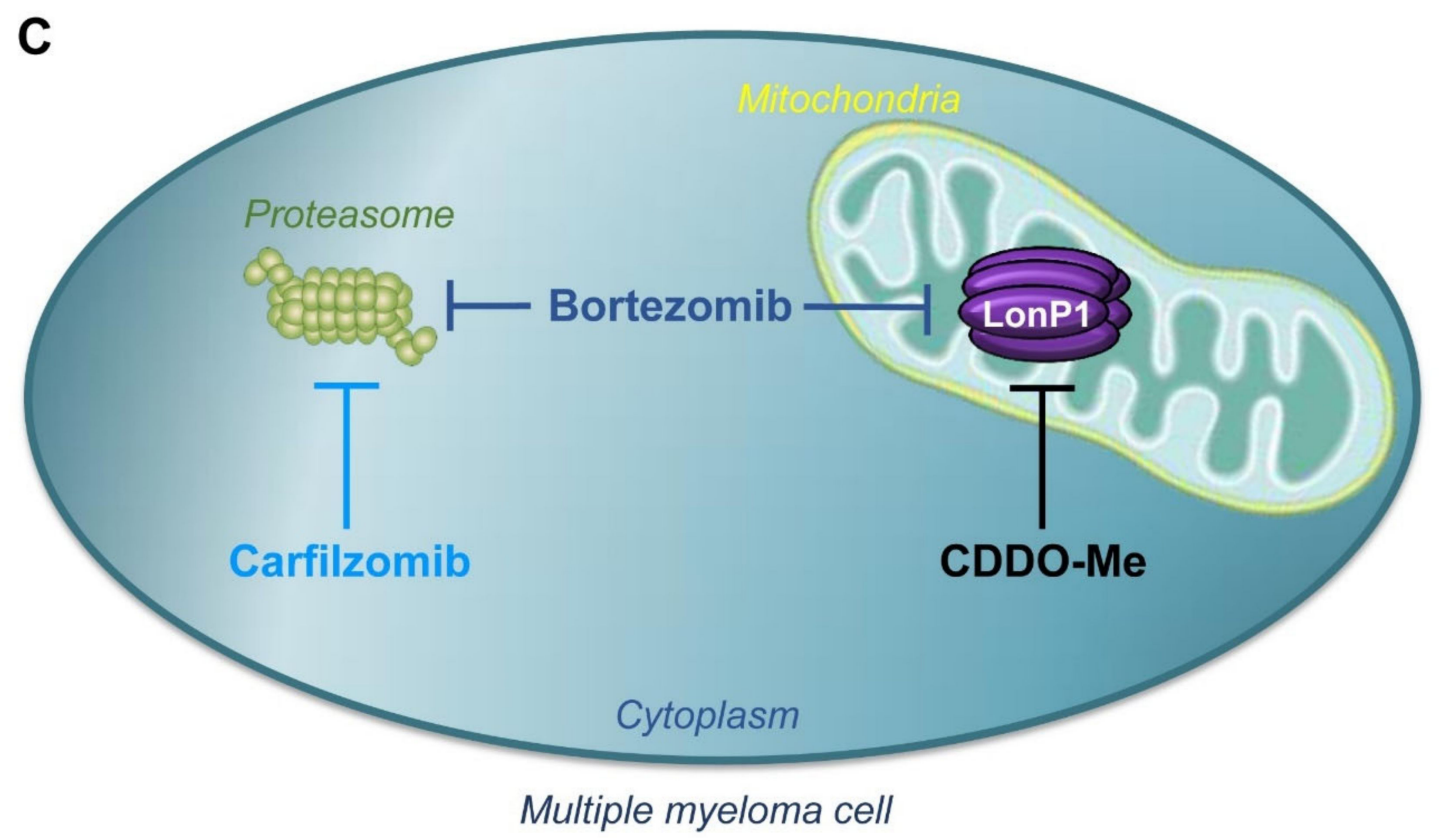
Additive and synergistic effects of LonP1 and proteasome inhibitors: (**A**) Determination of the cytotoxic range of the LonP1 inhibitor CDDO-Me in MM.1S cells in acute toxicity assays (24 h treatment). We used the more potent methyl-ester derivative of CDDO (CDDO-Me) in all experiments. Concentrations of up to 300 nM had no toxic effect as measured by XTT assay (shown here) and by acridine orange and propidium iodide staining (not shown). The individual data points indicate averages and the error bars standard deviations. Applying the LonP1-specific inhibitor CDDO-Me at sublethal concentrations (300 nM) in MM.1S cells strongly increased the cytotoxic effect of carfilzomib, but only mildly increased the effect of bortezomib; (**B**) Isobologram analysis indicates synergy between CDDO-Me and carfilzomib, but only additive effects between CDDO-Me and bortezomib. These experiments were performed in MM.1S cells and confirmed with adjusted concentrations in MOLP-8 cells. * *p* < 0.05 by unpaired Student’s two-tailed *t*-test. The individual data points indicate averages and the error bars standard deviations; (**C**) Model of proposed drug effects on the proteasome and on LonP1.

The essential protease LonP1 forms a homohexamer that resides in the mitochondrial matrix [29,32,33]. The synthetic triterpenoid CDDO-Me is a specific inhibitor of this protease without effect on the proteasome [34,35]. CDDO-Me displays potent anticancer activity in preclinical studies of B cell lymphomas. We determined the lethal concentration at which 50% of multiple myeloma cells died in vitro (LC_50_) following CDDO-Me treatment of two multiple myeloma cell lines [36]. The LC_50_ was determined to be 721 nM for MM.1S and 548 nM for MOLP-8 cells (Figure 4A. If carfilzomib is truly a proteasome-selective drug and LonP1 and the proteasome both cooperate in protein homeostasis, then we hypothesize that dual inhibition of these proteases should result in significantly increased cytotoxicity. The highly potent and proteasome-specific drug carfilzomib in combination with the LonP1-inhibitor CDDO-Me should synergistically enhance protein stress within multiple myeloma cells, while bortezomib, a baseline inhibitor of LonP1 and less potent proteasome inhibitor, may not derive a synergistic benefit with addition of CDDO-Me. We applied the Chou-Talalay approach and calculated the combination index of carfilzomib with CDDO-Me or bortezomib with CDDO-Me [37,38]. As predicted, the combination index of carfilzomib and CDDO-Me was <1, indicating synergy between the two drugs. In contrast, the combination index of bortezomib and CDDO-Me was =1, indicating an additive relationship between both drugs (Figure 4B). These pharmacological experiments confirm reports that bortezomib functionally acts as a dual inhibitor of both the proteasome and LonP1 [20]. Thus, adding the LonP1 inhibitor CDDO-Me only modestly increases the cytotoxicity of bortezomib. In contrast, carfilzomib only inhibits the proteasome, and the cytotoxic efficacy of carfilzomib can be drastically increased by combining it with the LonP1 inhibitor CDDO-Me (Figure 4C). Similarly, we observed that the combination of sublethal concentrations of CDDO-Me with proteasome inhibitors drastically increased the level of caspase 3 cleavage (Supplementary Figure S2). This suggests that a synergistic effect between these two drugs might enhance the degree of intrinsic apoptosis.

One concern is that CDDO-Me can exhibit off-target effects [39]. In particular, CDDO-Me has been described as inhibiting the NF-κB pathway, which is relevant for growth of myeloma cells [40,41,42]. However, an analysis of NF-κB target genes shows no repression of this pathway in MM.1S and MOLP-8 cells upon treatment with CDDO-Me (Supplementary Figure S3). This drug is therefore unlikely to increase cell death in combinatorial treatment due to inhibition of the NF-κB pathway.

These experiments do not address whether LonP1 and the proteasome act in the same functional pathways. If the mitochondrial protease LonP1 can aid the proteasome and engage in the degradation of extramitochondrial cytosolic proteins, then the overexpression of LonP1 should temper the effects of proteasome inhibition. The clinical and pharmacogenomic data from the Mulligan et al. study (Figure 1B) show aggressive growth of multiple myeloma under proteasome inhibition when LonP1 is highly expressed. However, these results can be differently interpreted: patients with multiple myeloma cells expressing high levels of LonP1 are more resistant to bortezomib (causality), or higher expression of LonP1 is an epiphenomenon in cancers that show more aggressive growth under therapy (correlation). To distinguish between these two possibilities, we transduced the clonal MM.1S and MOLP-8 multiple myeloma cell lines with mock virus or with a lentivirus to overexpress LonP1 and tested the impact of this protease on proteasome inhibition.

**Figure 5:**
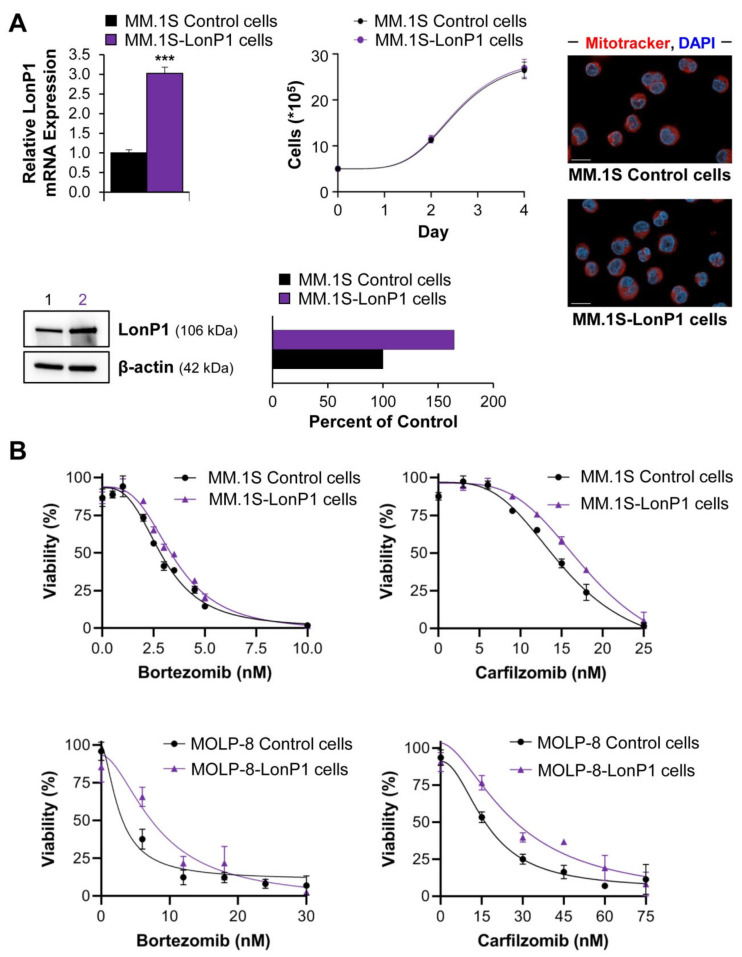

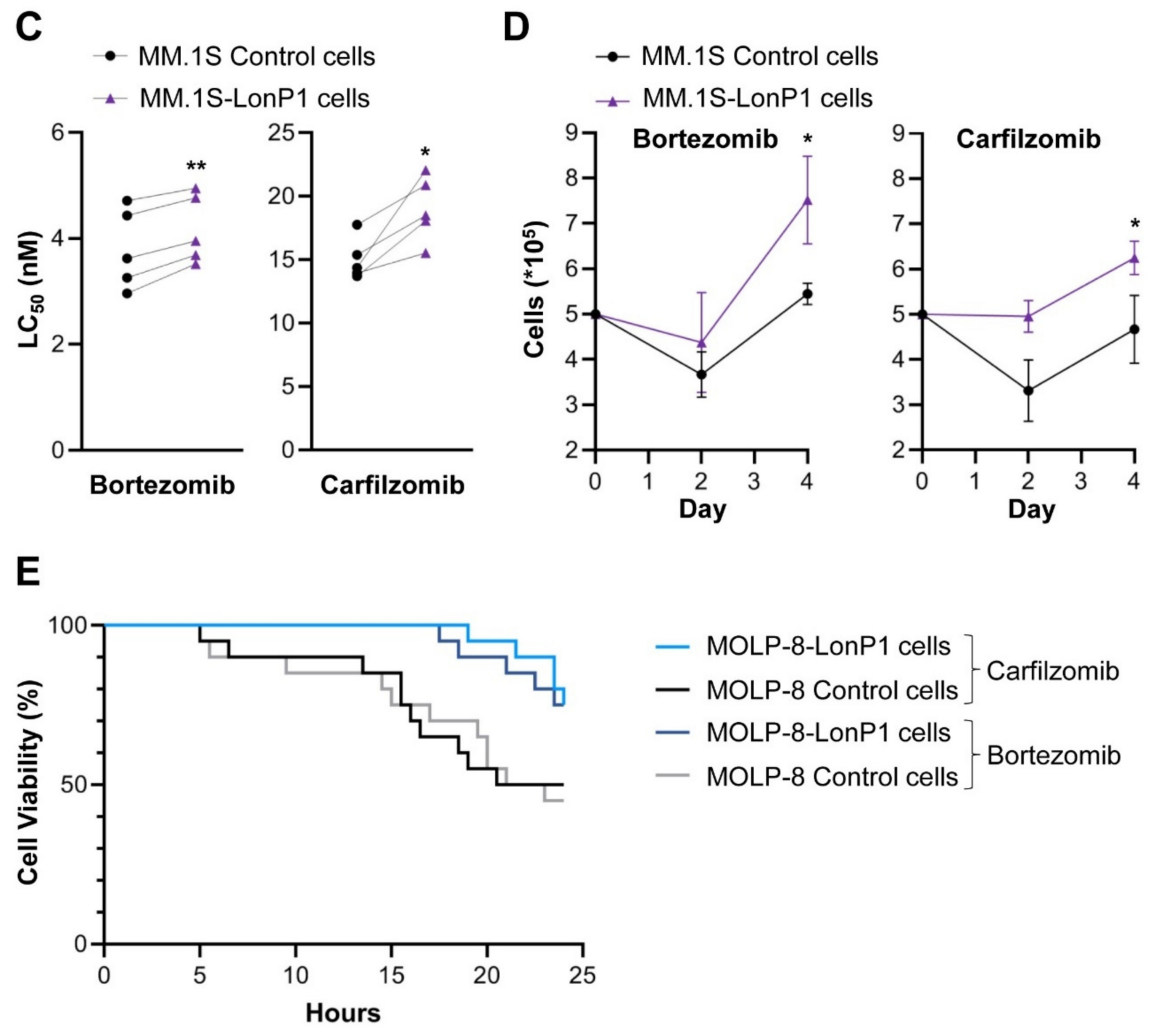
Effect of LonP1 overexpression on proteasome inhibition: (**A**) MM.1S cells were stably transduced with lentivirus to overexpress human LonP1. Compared to mock-transduced cells, LonP1 expression was 2.5-fold increased at the RNA level. *** *p* < 0.001 by unpaired Student’s two-tailed *t*-test. Based on immunoblot, LonP1 expression was 1.6-fold up-regulated at the protein level. Lane 1 contains lysate from MM.1S control cells, and lane 2 the equal amount of lysate of LonP1-transduced MM.1S cells. We confirmed the increase in LonP1 protein expression by quantitative immunofluorescence microscopy in MM.1S and MOLP-8 cells (Supplementary Figure S4). LonP1-transduced cells grew at a comparable rate to wild-type cells and show similar morphology following staining with Mitotracker Red CMXRos (red) and DAPI (blue). Scale bar = 10 µm. The individual data points indicate averages and the error bars standard deviations; (**B**) Shown are exemplary viability curves of MM.1S and MOLP-8 cells with or without overexpression of LonP1. Cells with higher levels of LonP1 showed a right shift, indicating partial resistance to drug treatment. The individual data points indicate averages and the error bars show standard deviations; (**C**) An analysis of five independent experiments as shown in Figure 5B ndicates that the LC_50_ of bortezomib and carfilzomib in acute toxicity assays (24 h) was increased by 10% and 25%, respectively (*p* = 0.002 and *p* < 0.02 by paired Student’s two-tailed *t*-test) in MM.1S cells overexpressing LonP1; (**D**) Monitoring cell growth over the duration of four days in the presence of LC_50_ levels of bortezomib and carfilzomib (3.3 nM and 13.3 nM, respectively) showed that LonP1-transduced MM.1S cells grew significantly better (* *p* < 0.05 and ** *p* < 0.01 determined by unpaired Student’s two-tailed *t*-test at day 4). Since carfilzomib does not directly inhibit LonP1, these results suggest that the mitoprotease supports functions otherwise performed by the proteasome. The individual data points indicate averages and the error bars show standard deviations; (**E**) Live single-cell tracking of 20 MOLP-8 cells per genotype and treatment (80 cells in total). Immobilized cells were continuously analyzed by brightfield microscopy over the course of 24 h to determine viability in the presence of 12 nM bortezomib or 30 nM carfilzomib. Untreated control cells were 100% viable. LonP1-overexpressing MOLP-8 cells were significantly more resistant to treatment (*p* < 0.05 based on both Log-rank test and Gehan–Breslow–Wilcoxon test).

LonP1 is an essential protein and genetic knockouts are not viable. Also, strong overexpression was toxic in several cell lines that we tested. However, MM.1S and MOLP-8 cells tolerated a modest two- to three-fold up-regulation of the mitoprotease without impacting overall growth or cell morphology (Figure 5A). The acute cytotoxicities of bortezomib and carfilzomib were significantly reduced by 10% and by 25%, respectively, when myeloma cells overexpressed LonP1 (Figure 5B,C). The effects on cell growth in the presence of these drugs were even stronger. LonP1-transduced MM.1S cells displayed >30% higher numbers after four days of growth in the presence of bortezomib or carfilzomib at LC_50_ concentrations (Figure 5D). Similar results were obtained for modified MOLP-8 cells (Figure 5B,E).

**Figure 6:**
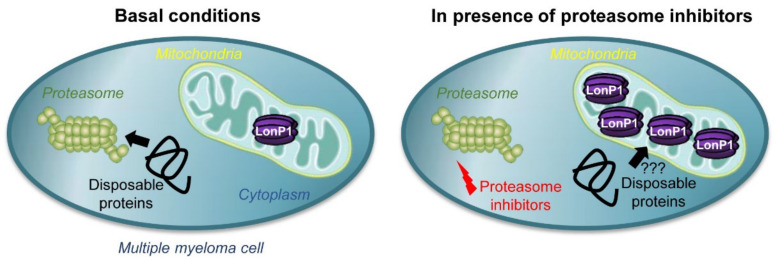
Model of LonP1 action in multiple myeloma.

These data clearly show that LonP1 can partially antagonize proteasome inhibition. While the results with bortezomib could be interpreted as direct antagonism toward the LonP1-directed inhibitory effect where LonP1 simply acts as a “drug sink” for bortezomib, carfilzomib does not bind to this mitoprotease. The fact that LonP1 counteracts carfilzomib therefore indicates that the proteasome and LonP1 engage in overlapping functions, and that LonP1 can to some degree compensate for proteasome inhibition (Figure 6 and Graphical Abstract). Furthermore, LonP1 showed up to 15-fold differences in expression levels among primary multiple myeloma samples (Figure 2A), indicating that this mitoprotease might contribute to clinically relevant resistance mechanisms or the emergence of relapsed/refractory multiple myeloma.

## 3. Discussion

Despite promising advances in drug development and clinical therapies, multiple myeloma remains an incurable cancer, and all current treatment regimens eventually lead to drug resistance. The nature of resistance likely involves several different mechanisms, and mitochondria have been identified as a central organelle that contributes to refractory disease upon proteasome inhibition [10]. However, the molecular mechanisms that are involved in the emergence of resistant cancer clones are ill-defined.

Since their approval for the treatment of multiple myeloma, proteasome inhibitors have become the drugs of choice in several first-line chemotherapy combinations. The proteasome is a barrel-shaped threonine protease complex containing the active sites PSMB1, PSMB2, and PSMB5. Pharmacological inhibition of the PSMB5 subunits by drugs such as bortezomib or carfilzomib leads to the accumulation of poly-ubiquitinated proteins that cannot be properly degraded by the proteasome [43]. LonP1 is a serine protease that primarily resides in the mitochondrial matrix and has a similar shape as the core proteasome. LonP1 is encoded in the nucleus and imported into the organelle following translation by cytosolic ribosomes. Even though the main effects of mitochondrial proteases pertain to mitochondrial protein housekeeping, several lines of evidence suggest that mitoproteases can also engage in extra-mitochondrial protein quality control. First, the mitoprotease HTRA2/OMI can leak out from the intermembrane space to degrade cytosolic proteins [18]. Second, the cancer-dependency map, a collection of large-scale loss-of-function screens, provides evidence that LonP1 and proteasomal subunits converge in a common pathway [44]. Third, a study in yeast discovered that the LONP1 ortholog PIM1 can effectively degrade cytosolic protein aggregates that cannot be removed by the proteasome [19].

In light of these studies, a concept emerges that mitochondria are more than metabolic hubs. Equipped with powerful proteases, they are capable of roaming the cytoplasm to engulf proteins and contribute to protein quality control outside their own boundaries. Such a model would also explain why the more potent irreversible second-generation drug carfilzomib only shows disappointing results when used for the treatment of relapsed/refractory multiple myeloma that is resistant to the reversible first-generation inhibitor bortezomib [45]. The enhanced clinical effect of carfilzomib compared to bortezomib is likely due to the more forceful inhibition of the proteasome by the former drug [43,46]. We hypothesize that the effectiveness of carfilzomib in particular would benefit from combining it with a synergistic LonP1 inhibitor.

Novel functions of LonP1 continue to be discovered. For instance, a recent publication describes chaperone-like activity, suggesting that LonP1 might alleviate proteostatic stress through refolding of proteins in addition to degradation [47]. Also, future studies are needed to determine the specificity of cytosolic protein recognition by mitochondrial proteases, to investigate the role of ubiquitination, and to establish the location of this interaction. Previous evidence suggests that proteases can leak out of mitochondria, even though this activity seems more likely for proteases in the outer compartments. For matrix proteases such as LonP1, it is possible that they act in the cytosol prior to mitochondrial import. Although not mutually exclusive, active import of cytosolic proteins into the mitochondrial matrix may be required based on studies in yeast [19]. This raises the prospect that inhibiting certain mitochondrial transporters may synergize in chemotherapies that aim to perturb cytosolic or nuclear protein quality control. It certainly makes the ancient LonP1 protease, an enzyme that is conserved in all kingdoms of life, a promising new target for the treatment of multiple myeloma [48].

## 4. Materials and Methods

### 4.1. Survival Analysis

The survival times of participants in the study by Mulligan et al. [24] were compared based on individual expression levels of the 20 mitoproteases indicated in Figure 1A. Transcript data were obtained from Gene Expression Omnibus entry GSE9782. Differences in survival for patients whose expression levels for each of the target genes ranked in the top versus bottom quarter were deemed significant when <0.05 based on both Log-rank test and Gehan–Breslow–Wilcoxon test (calculated with GraphPad Prism version 9.0).

### 4.2. Drug Treatments

The proteasome inhibitors bortezomib and carfilzomib were obtained from Selleckchem (Houston, TX, USA, #PS-341 and #PR-171, respectively). Lactacystin was purchased from Cayman Chemical (Ann Arbor, MI, USA, #70980) and 2-Cyano-3,12-dioxo-oleana-1,9(11)-dien-28-oic acid methyl ester (CDDO-Me) was purchased from Sigma-Aldrich (St. Louis, MO, USA, #SMB00376).

### 4.3. Transcript Analysis

Expression levels of LONP1 and OSGEPL1 in primary myeloma cells were compared to all other transcripts in 747 patients. The data was part of the CoMMpass study by the Multiple Myeloma Research Foundation (MMRF), release IA10 [25] (https://research.themmrf.org; access date 20 October 2020). Gene expression was normalized with Cufflinks as FPKM (fragments per kilobase million) [49]. The gene-ontology analysis was performed with the DAVID database [26]. Mitochondrial protease expression levels in MM.1S myeloma cell lines following treatment with 6 µM lactacystin or 60 nM bortezomib for six hours were compared to mock-treated cells (DMSO at 0.1% *v*/*v*). RNA was extracted with the RNeasy mini kit with RNAse-free DNaseI on column treatment (Qiagen, Hilden, Germany, #74134 & #79254) and quantified by paired-end RNA-sequencing on an Illumina HiSeq2500, as previously published [50]. RNA-seq datasets have been deposited with the Gene Expression Omnibus (GEO accession number GSE166122). The heatmap was constructed with GraphPad Prism version 9.0 following normalization of data with Cufflinks and Cuffdiff version 2.1.1 [49]. RT-qPCR was performed with the iTaq Universal SYBR Green One-Step Kit (Bio-Rad, Hercules, CA, USA, #1725151) according to manufacturer’s instructions on a Bio-Rad CFX96 real time PCR instrument. The following primers were used:

- β-actin (F-5’-CATGTACGTTGCTATCCAGGC; R-5′-CTCCTTAATGTCACGCACGAT);
- LONP1 (F-5′-ATGGAGGACGTCAAGAAACG; R-5′-GATCTCAGCCACGTCAGTCA);
- OSGEPL1 (F-5′-AAAACAGGTGGGATTGTTCCTC; R-5′-AGTAAGTGCATGAGCCTCCAT);
- CD40 (F-5′-ACTGAAACGGAATGCCTTCCT; R-5′-CCTCACTCGTACAGTGCCA);
- COX2 (F-5′-TAAGTGCGATTGTACCCGGAC; R-5′-TTTGTAGCCATAGTCAGCATTGT);
- ICAM-1 (F-5′-ATGCCCAGACATCTGTGTCC; R-5′-GGGGTCTCTATGCCCAACAA);
- IL-6 (F-5′-CCTGAACCTTCCAAAGATGGC; R-5′-TTCACCAGGCAAGTCTCCTCA);
- IRF3 (F-5′-AGAGGCTCGTGATGGTCAAG; R-5′-AGGTCCACAGTATTCTCCAGG).

### 4.4. Computational Modeling

In silico modeling was done using the LSQ and SSM superpose routines, and manual fitting in Coot [51] using the atomic coordinates of human mitochondrial LonP1 (PDB: 7KSM) [30], and the LonA protease domain in complex with bortezomib (PDB: 4YPM) [29]. Atomic coordinates for carfilzomib were derived from PDB: 4QW6.H [31]. Carfilzomib and bortezomib were aligned using C47 of carfilzomib and B26 of bortezomib as anchor. All structure figures were generated using the PyMOL Molecular Graphics System version 1.7.0.3 (Schrödinger, LLC, New York City, NY, USA).

### 4.5. Cytotoxicity Assays

MM.1S and MOLP-8 cells were obtained from the repositories ATCC (#CRL 1974) and DSMZ (#ACC 569), respectively. Cells were grown in humidified incubators with 5% CO_2_ in recommended growth medium and regularly tested for mycoplasma contamination. For acute cytotoxicity assays, 30,000 cells were seeded in fresh medium per well in 96-well plates with the indicated final concentration of drugs at 100 μL total volume. After 24 h, cell viability was measured with the XTT cell proliferation kit (Roche, Indianapolis, IN, USA, #11465015001) and a Bio-Tek (Winooski, VT, USA) PowerWave XS microplate reader using the 450 nm (readout) and 630 nm (reference) absorbance filters with the Gen5 version 2.09 software. For long-term cytotoxicity assays, 500,000 cells per well were plated in fresh medium in 6-well plates in the absence or presence of LC_50_ concentrations of bortezomib (3.3 nM) or carfilzomib (13.3 nM). At days 2 and 4 after careful mixing, 100 μL aliquots of cells per condition were removed, and viable cells were counted based on acridine orange and propidium iodide staining (Nexcelom Bioscience, #CS2-0106) with a Cellometer Auto 2000 (Nexcelom Bioscience, Lawrence, MA, USA). Medium or drugs were not replenished during this assay. All experiments were confirmed in >3 biological replicates, and all measurements were taken as triplicates or quadruplicates.

### 4.6. Generation of Transgenic MM.1S and MOLP-8 Cells

MM.1S and MOLP-8 cells were infected with lentivirus-encoding human LonP1, cloned into the vector pLVX-EF1α (Takara Biosciences, Nojihigashi, Japan, #631982), and sorted to 100% purity by FACS following ZsGreen1 expression with a BD FACSAria II cell sorter. A virus containing the empty pLVX construct was used as control.

### 4.7. Microscopy

Mitochondria were stained using MitoTracker Red CMXRos (Thermo Fisher, Waltham, MA, USA, #M7512). In short, one million MM.1S control or LonP1-transduced cells were taken up in 0.4 mL fresh medium containing 1 μM MitoTracker Red CMXRos and applied onto glass slides pretreated with Cell-Tak (Corning, Corning, NY, USA, #354240) for 30 min in a humidified incubator with 5% CO_2_. Afterwards, unsettled cells were washed off with PBS and adherent cells were permeabilized and fixed with PBS, 0.2% Triton-X100, and 4% formaldehyde solution (Thermo Fisher, Waltham, MA, USA, #28906) for 10 min at room temperature. After washing with PBS, cells were mounted and counterstained with DAPI (Invitrogen, Waltham, MA, USA, #P36935). Microscopy was performed with a Zeiss CD7 live cell imager at 100x magnification, and pictures were processed for deconvolution with identical settings using the ZEN Pro 3.0 software. Immunofluorescence microscopy was performed on Cell-Tak-immobilized myeloma cells, following fixation and permeabilization as described above. We used antibodies against cleaved caspase 3 (Cell Signaling Technology, Danvers, MA, USA, #9664), against LonP1 (Abcam, Cambridge, MA, USA, #ab224316), and against β-tubulin (Cell Signaling Technology, Danvers, MA, USA, #86298) at 1:500 dilutions overnight in the fridge after blocking with 10% (*w/v*) BSA. Secondary antibodies were employed at room temperature for one hour at 1:1000 dilutions (Invitrogen, Waltham, MA, USA, #A11005 and #A11034). Consistent exposure and intensity settings were used for quantitative microscopy. Fourteen z-stacks were taken at 100x magnification, and deconvolution was performed using the nearest-neighbor approach, followed by orthogonal projection at maximum settings (ZEN Pro 3.0). Brightness was quantified for the specified channel using the FIJI software (https://imagej.net/Fiji/Downloads; download date: 23 May 2019) and with DAPI as reference. Settings were adjusted to MM.1S and MOLP-8 cell lines but left consistent within each cell type. Live single-cell tracking was performed within the humidified incubation unit of the CD7 microscope at 37 °C with 5% CO_2_ on Cell-Tak treated optical 24 well plates at 20x magnification (Greiner, Frickenhausen, Germany # 662892). No cell toxicity was observed in DMSO mock-treated MOLP-8 cells during the 24 h observation time. Pictures were taken every 30 min.

### 4.8. Immunoblotting

Proteins were separated by SDS-PAGE under reducing conditions and then semi-dry transferred onto polyvinylidene difluoride (PVDF) membranes for 7 min using a Bio-Rad Turbo Transfer system. Then, PVDF membranes were blocked with 5% milk in TBST. Western-blot analysis against LonP1 was carried out using a rabbit polyclonal anti-LonP1 antibody (Abcam, Cambridge, MA, USA, #ab224316, diluted 1:1,000, overnight at 4 °C) with β-actin rabbit monoclonal antibody (Cell Signaling Technology, #8457, employed 1:1,000) as a loading control. Following incubation with HRP-conjugated goat antirabbit secondary antibody (Abcam, Cambridge, MA, USA, #ab6721 at 1:6000 dilution for 75 min), HRP-conjugated proteins were detected with the SuperSignal West Pico PLUS chemiluminescent substrate (Thermo Scientific, Waltham, MA, USA, #34577) and visualized using the Bio-Rad (Hercules, CA, USA) ChemiDoc imaging system.

## 5. Conclusions

In this study, we provide evidence that the mitochondrial matrix protease LonP1 causes partial resistance to proteasome inhibition in multiple myeloma. In particular, treatment with more specific second-generation proteasome inhibitors such as carfilzomib may benefit from a dual approach and combinatorial use with LonP1 inhibitors.

## Data Availability

The RNA-seq data presented in this study is openly available at the Gene Expression Omnibus data repository (GEO accession number GSE166122) at https://www.ncbi.nlm.nih.gov/geo/. Publicly available transcriptome datasets from the CoMMpass study by the Multiple Myeloma Research Foundation (MMRF) can be accessed here: https://research.themmrf.org. Publicly available transcriptome and clinical data from the study by Mulligan et al. [24] are available under GEO accession number GSE9782.

## Supplementary Materials

The following are available online at https://www.mdpi.com/2072-6694/13/4/843/s1, Table S1: Excel file listing genes that correlate and anti-correlate with LONP1 and OSGEPL1 in primary multiple myeloma cells, Figure S1: Proteasome inhibition increases LonP1 expression in MM.1S and MOLP-8 cells: Immuno-fluorescence microscopy of multiple myeloma cell lines following 24 hours of treatment with 1 nM bortezomib or 5 nM carfilzomib. Pictures were taken at 100× magnification (scale bar = 10 µm), Figure S2: Increased apoptosis in MM1.S cells treated with a combination of CDDO-Me and proteasome inhibitors: Cells were treated for 24 hours with sublethal concentrations of CDDO-Me (300 nM) and/or 3 nM bortezomib or 15 nM carfilzomib. Combined treatment shows strikingly higher levels of apoptosis, as indicated by immuno-fluorescence detection of cleaved caspase 3. Pictures were taken at 20× magnification (scale bar = 100 µm) for panoramic view and 100× (scale bar = 10 µm) for depiction of an apoptotic cell, Figure S3: The NF-κB pathway does not appear to be suppressed by CDDO-Me treatment: MM.1S cells were treated for six hours with 500 nM CDDO-Me, 10 nM bortezomib, or 20 nM carfilzomib. MOLP-8 cells were treated for six hours with 500 nM CDDO-Me, 20 nM bortezomib, or 20 nM carfilzomib. RNA was extracted and the expression of NF-κB target genes was assayed by RT-qPCR. * *p* < 0.05, ** *p* < 0.01, and *** *p* < 0.001 by unpaired Student’s two-tailed *t*-test. Not all target genes tested were expressed in MOLP-8 cells. Target genes are based on a resource by the Gilmore lab: https://www.bu.edu/nf-kb/gene-resources/target-genes/, Figure S4: Higher LonP1 expression in stably transduced MM.1S and MOLP-8 cells: Cells infected with a lentivirus encoding human LONP1 show significantly higher levels of expression of this mitoprotease. Top: representative immuno-fluorescence pictures at 100× magnification (scale bar = 10 µm). Bottom: quantification of LonP1 signal using DAPI as reference, as measured in 20 randomly chosen cells. *** *p* < 0.001 by unpaired Student’s two-tailed *t*-test.
