# Supplementary material for: The Mitochondrial Protease LonP1 Promotes Proteasome Inhibitor Resistance in Multiple Myeloma
Source: Cancers (Basel). 2021 Feb 17;13(4):843. doi: 10.3390/cancers13040843 (PMC7922145; doi:10.3390/cancers13040843)
Supplement: Supplementary file 1 [file cancers-13-00843-s001.zip › cancers-1080531-supplementary materials.docx]

The mitochondrial protease LonP1 promotes proteasome inhibitor resistance in multiple myeloma

Laure Maneix ^1,2,3,4^, Melanie A. Sweeney ^1,2,3,4^, Sukyeong Lee ^5^, Polina Iakova ^1,2,3^,

Shannon E. Moree ^1,2,3,4^, Ergun Sahin ^2^, Premal Lulla ^4,6,7^, Sarvari V. Yellapragada ^6,7,8^,

Francis T.F. Tsai ^1,5,9^ and Andre Catic ^1,2,3,4,7,8,^*

Supplementary Figures:


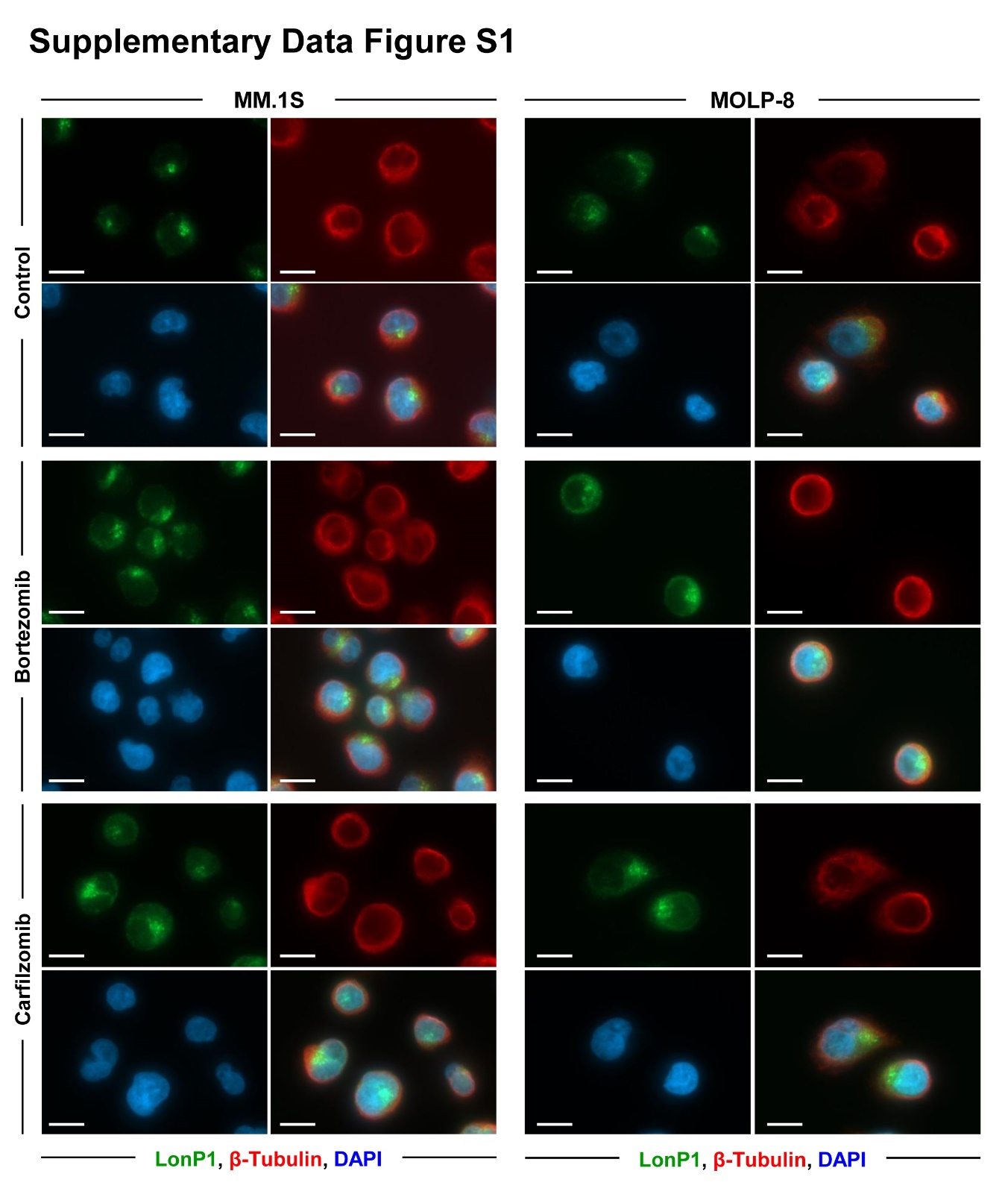


**Figure S1.** Proteasome inhibition increases LonP1 expression in MM.1S and MOLP-8 cells: Immuno-fluorescence microscopy of multiple myeloma cell lines following 24 hours of treatment with 1 nM bortezomib or 5 nM carfilzomib. Pictures were taken at 100x magnification (scale bar=10 µm).


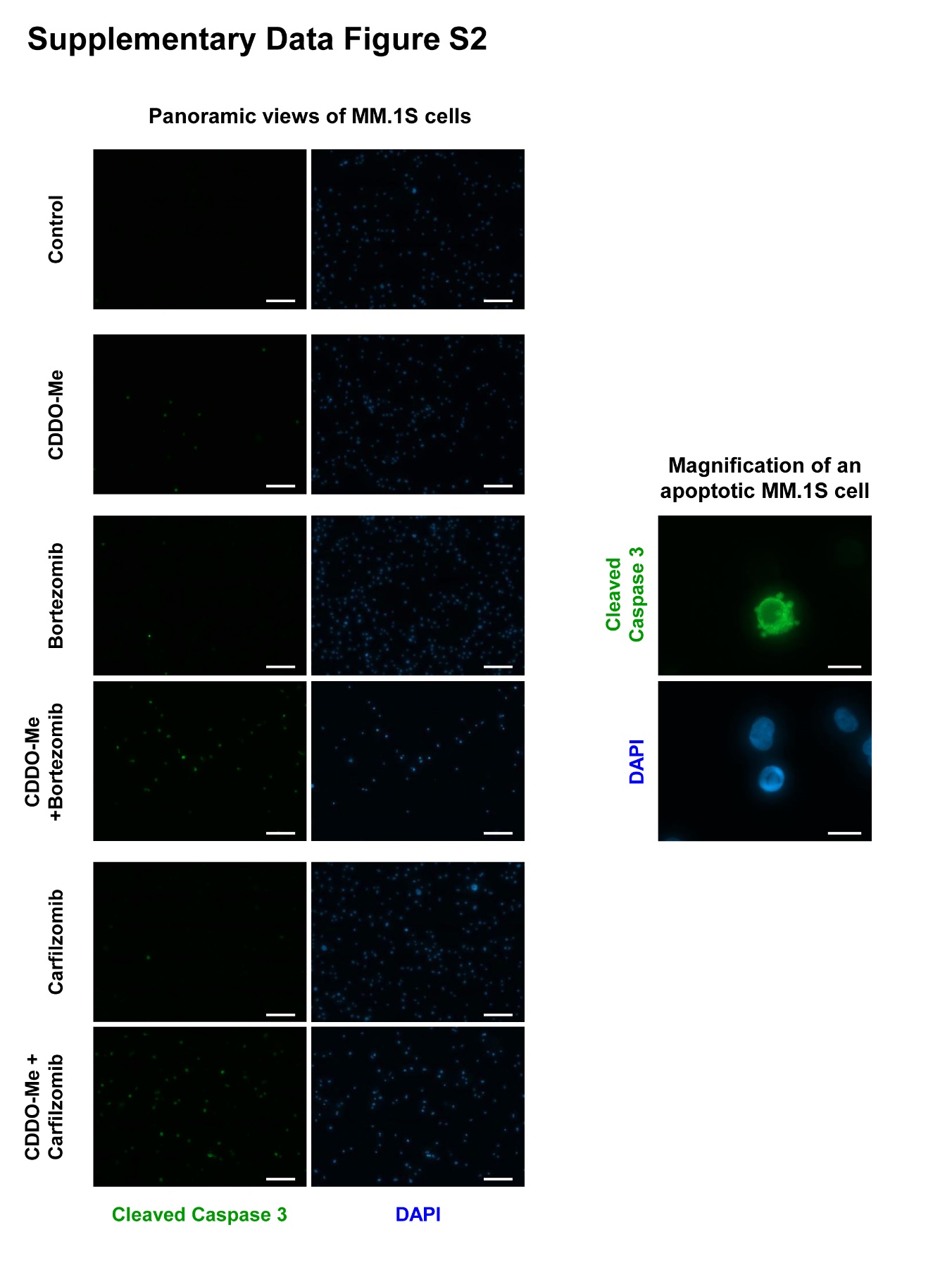


**Figure S2.** Increased apoptosis in MM1.S cells treated with a combination of CDDO-Me and proteasome inhibitors: Cells were treated for 24 hours with sublethal concentrations of CDDO-Me (300 nM) and/or 3 nM bortezomib or 15 nM carfilzomib. Combined treatment shows strikingly higher levels of apoptosis, as indicated by immuno-fluorescence detection of cleaved caspase 3. Pictures were taken at 20x magnification (scale bar=100 µm) for panoramic view and 100x (scale bar=10 µm) for depiction of an apoptotic cell.

**
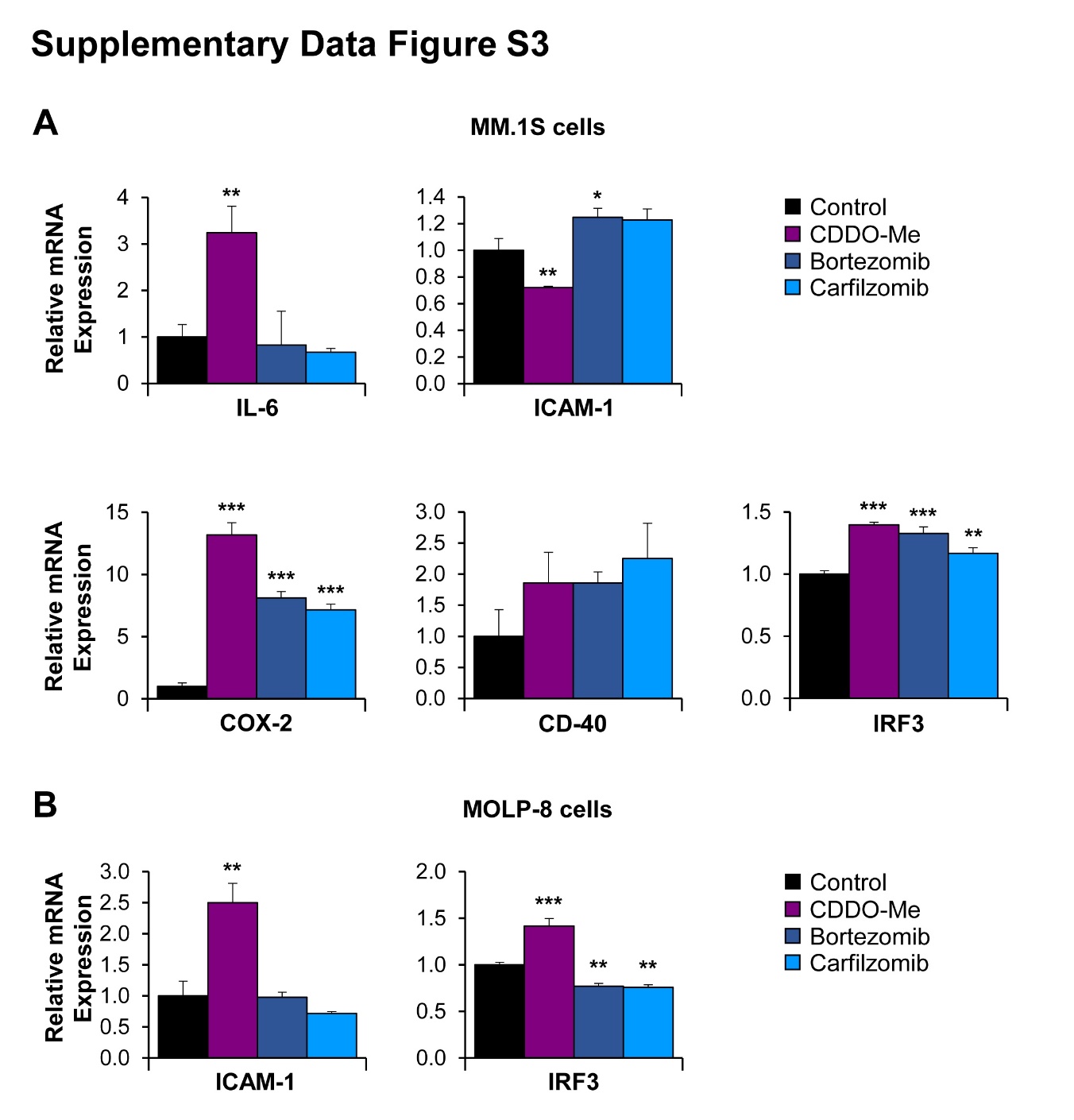
**

**Figure S3.** The NF-κB pathway does not appear to be suppressed by CDDO-Me treatment: MM.1S cells were treated for six hours with 500 nM CDDO-Me, 10 nM bortezomib, or 20 nM carfilzomib. MOLP-8 cells were treated for six hours with 500 nM CDDO-Me, 20 nM bortezomib, or 20 nM carfilzomib. RNA was extracted and the expression of NF-κB target genes was assayed by RT-qPCR. *p < 0.05, **p < 0.01, and ***p<0.001 by unpaired Student’s two-tailed t-test. Not all target genes tested were expressed in MOLP-8 cells. Target genes are based on a resource by the Gilmore lab: [https://www.bu.edu/nf-kb/gene-resources/target-genes](about:blank)/.


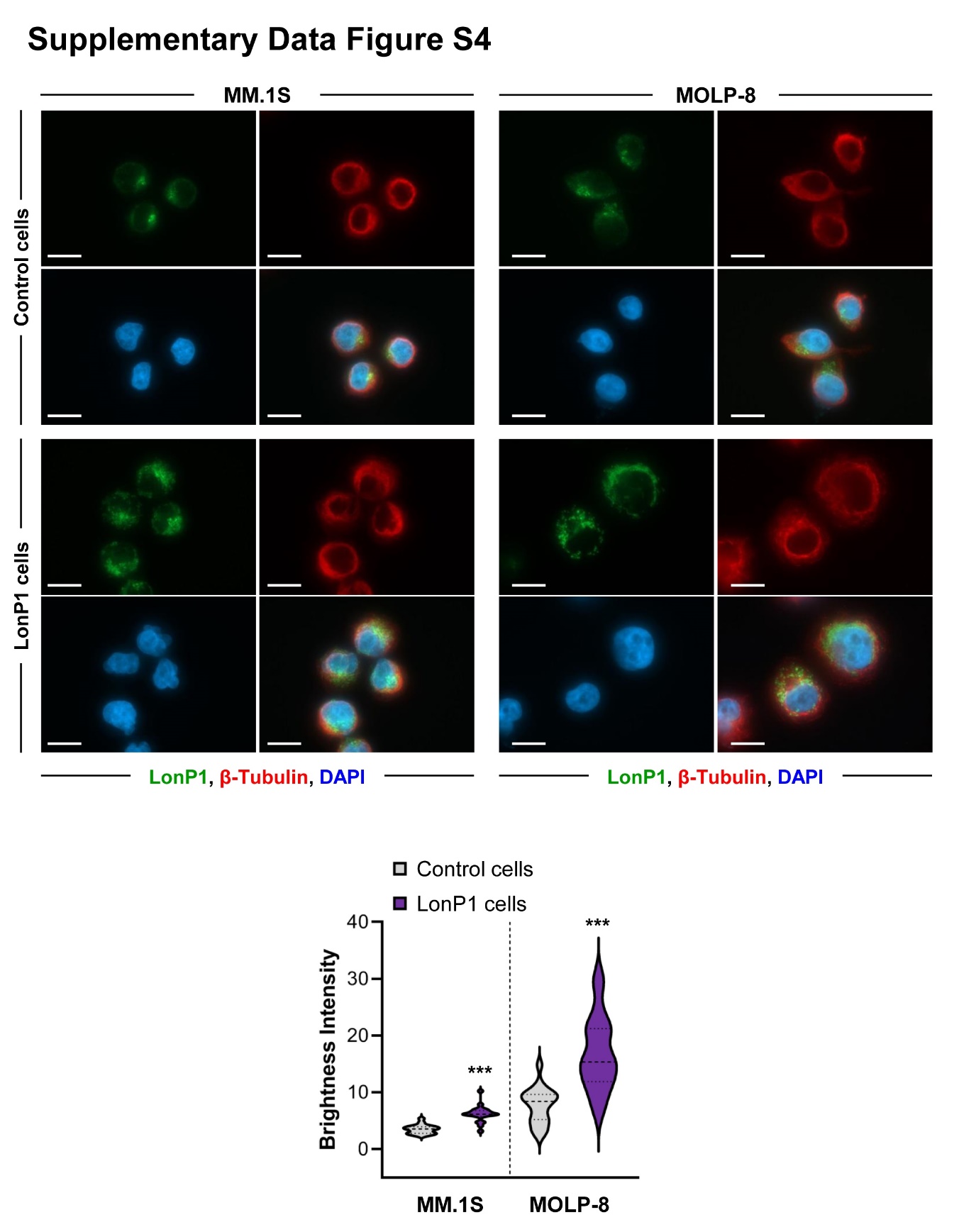


**Figure S4.** Higher LonP1 expression in stably transduced MM.1S and MOLP-8 cells: Cells infected with a lentivirus encoding human LONP1 show significantly higher levels of expression of this mitoprotease. Top: representative immuno-fluorescence pictures at 100x magnification (scale bar=10 μm). Bottom: quantification of LonP1 signal using DAPI as reference, as measured in 20 randomly chosen cells. ***p<0.001 by unpaired Student’s two-tailed t-test.
